# Natural Bioactive Compounds from Fungi as Potential Candidates for Protease Inhibitors and Immunomodulators to Apply for Coronaviruses

**DOI:** 10.3390/molecules25081800

**Published:** 2020-04-14

**Authors:** Nakarin Suwannarach, Jaturong Kumla, Kanaporn Sujarit, Thanawat Pattananandecha, Chalermpong Saenjum, Saisamorn Lumyong

**Affiliations:** 1 Research Center of Microbial Diversity and Sustainable Utilization, Chiang Mai University, Chiang Mai 50200, Thailand; jaturong_yai@hotmail.com (J.K.); kanaporn_s@rmutt.ac.th (K.S.); scboi009@gmail.com (S.L.); 2 Department of Biology, Faculty of Science, Chiang Mai University, Chiang Mai 50200, Thailand; 3 Division of Biology, Faculty of Science and Technology, Rajamangala University of Technology Thanyaburi, Thanyaburi, Pathumthani 12110, Thailand; 4 Department of Pharmaceutical Sciences, Faculty of Pharmacy, Chiang Mai University, Chiang Mai 50200, Thailand; thanawat.pdecha@gmail.com (T.P.); chalermpong.saenjum@gmail.com (C.S.); 5 Academy of Science, The Royal Society of Thailand, Bangkok 10300, Thailand

**Keywords:** antiviral agents, drug discovery, coronaviruses, fungal metabolites, immunomodulatory agents, natural products

## Abstract

The inhibition of viral protease is an important target in antiviral drug discovery and development. To date, protease inhibitor drugs, especially HIV-1 protease inhibitors, have been available for human clinical use in the treatment of coronaviruses. However, these drugs can have adverse side effects and they can become ineffective due to eventual drug resistance. Thus, the search for natural bioactive compounds that were obtained from bio-resources that exert inhibitory capabilities against HIV-1 protease activity is of great interest. Fungi are a source of natural bioactive compounds that offer therapeutic potential in the prevention of viral diseases and for the improvement of human immunomodulation. Here, we made a brief review of the current findings on fungi as producers of protease inhibitors and studies on the relevant candidate fungal bioactive compounds that can offer immunomodulatory activities as potential therapeutic agents of coronaviruses in the future.

## 1. Introduction

Coronaviruses (CoVs) are a large group of enveloped viruses with non-segmented, single-strand, and positive-sense RNA genomes. CoVs are classified in the family Coronaviridae of the order Nidovirales. Notably, CoVs have been identified as zoonotic viruses that can be transmitted between humans and animals and they are known to cause a wide range of infections. These infections can appear as symptoms that range from those of the common cold to much more fatal diseases, like respiratory syndrome, as well as enteric and central nervous system diseases. Two highly pathogenic microorganisms with approximately 30,000 nucleotides, the Severe Acute Respiratory Syndrome (SARS-CoV, or SARS) and the Middle East Respiratory Syndrome (MERS-CoV, or MERS), have resulted in regional and global outbreaks. In 2002, SARS originated in southern China, while MERS was first known to infect a patient in Saudi Arabia in 2012 [1,2,3]. A novel coronavirus, which was previously designated as SARS-CoV-2, was identified as a causal agent of pneumonia in Wuhan, a city in the Hubei Province, China, at the end of 2019 [4]. It has subsequently spread throughout China and elsewhere and it is now considered a global health emergency. In February 2020, the World Health Organization (WHO) labeled the disease SARS-CoV-2 or 2019-nCoV, which has been more commonly referred to as the coronavirus disease since its emergence in 2019. The mortality rate of SARS-CoV-2 infection has been seen to be around two percent in China, which is much less than the mortality rates of SARS-CoV and MERS-CoV infection [5]. However, it has caused global concern by its efficient human-to-human transmission, leading to its widespread outbreak in many countries around the world [4,5,6]. Currently, the WHO has referred to the SARS-CoV-2 outbreak as a “pandemic”, as emphasized the global risk of its spread and predictive elevates the risk of its impact to “very high”. Clinical practice guidelines and the treatment protocols of WHO and the Center for Disease Control and Prevention (CDC) for a patient infected with SARS-CoV-2 are similar to those of other viral causes of pneumonia. These include prompt supportive care, like oxygen therapy, fluid management, empiric antimicrobials (in case of sepsis), and others [5].

## 2. Protease Inhibitor Drugs for CoVs

**Figure 1:**
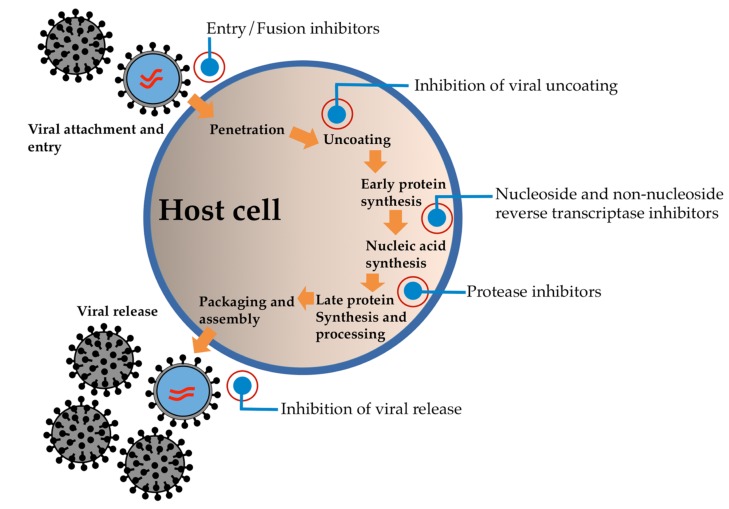
Major sites of antiviral drug action.

Generally, the inhibition of the viral replication process is of significant consideration in the treatment of viral infections (Figure 1). Protease is one of the necessary enzymes required for the replication, transcription, and maturation of a range of viruses [7,8]. Several studies have focused on the identification of an inhibitory target of protease, which is necessary for viral transcription/replication. Currently, the approved protease inhibitors are recognized as peptidomimetics and they are one of the first examples of a structure-based drug design that utilizes the structural information of inhibitor binding to the active site of viral protease [9,10].

Protease inhibitors play an important role in viral replication by selectively binding to viral proteases and blocking proteolytic cleavage of the protein precursors that are necessary for the production of infectious viral particles [7,8,11]. The papain-like protease (PL^pro^) and the 3-chymotrypsin-like protease (3CL^pro^, also known as the main proteases that are suitable targets for viral inhibitors) have been identified in CoVs, for which both proteases are believed to be essential in the role of viral replication and are considered to be attractive targets for antiviral therapeutics [12,13]. Numerous previous studies have identified compounds and drugs that can inhibit protease activity through docking/molecular dynamic experimentation and their inhibition activity on CoVs replication in the cell cultures of mice and in non-human primate (NHP) models, as has been previously reported [14]. On the other hand, human immunodeficiency virus type 1 (HIV-1) protease inhibitors (tipranavir, saquinavir, ritonavir, nelfinavir, lopinavir, indinavir, darunavir, atazanavir, and amprenavir) that have been approved for clinical applications by the Food and Drug Administration (FDA) are widely reported to be able to deactivate 3CL^pro^. Hence, they have been identified as potential drugs in the treatment of CoV infections. Lopinavir, atazanavir and indinavir have been identified as potential candidates as 3CL^pro^ inhibitors [15,16]. Furthermore, RNA-dependent RNA polymerase inhibitors, e.g., remdesivir and favilavir, have been used to effectively treat CoVs infections [16,17]. Remdesivir or chloroquine, glucocorticoids and the combined protease inhibitor lopinavir-ritonavir have been used to treat SARS-CoV and MERS-CoV infections [4,16,17,18].

Currently, there is a lack of an effective treatment or a vaccine to prevent SARS-CoV-2 infection. Parent compounds are being tested to prevent SARS-CoV-2 infection in in vitro or clinical studies-based SARS-CoV and MERS-CoV trials. Remdesivir or chloroquine has been highly effective in the inhibition of SARS-CoV-2 infection in vitro [18]. Lopinavir-ritonavir has been suggested to be one of the therapeutic agents of SARS-CoV-2 [17,19,20]. Hence, presently, there are no approved vaccines or drugs for SARS-CoV-2 infection. Potential candidates as targets for further in vitro and in vivo studies for SARS-CoV-2 prevention and treatment are 3CL^pro^, Spike, RNA-dependent RNA polymerase (RdRp), PL^pro^ and human angiotensin-converting enzyme 2 (human ACE2). Interestingly, the potential unitality of clinical drugs and natural products for the treatment of SARS-CoV-2 infection was studied while using computational methods. Specifically, 3CL^pro^ is recognized as an important target anti-SARS-CoV-2 drug that was designed and modified to play an important role in the maturation of SARS-CoV-2. Nelfinavir has been predicted as a potential inhibitor of the SARS-CoV-2 main protease, but has been used in treatment application [4]. Nevertheless, favilavir has been approved for use in the investigational therapy for SARS-CoV-2 infected patients in China [21]. The US patient infected with SARS-CoV-2 has been treated by supportive care and remdesivir, for which no adverse reactions were observed during administration [22,23]. In February 2020, the remdesivir treatment of SARS-CoV-2 was initiated in Wuhan and Beijing, China for clinical trials, but the safety and efficacy of remdesivir in the treatment process need to be fully evaluated [24]. Therefore, this study has summarized the current findings on the natural antiviral compounds that were obtained from fungi for the purpose of employing them as protease inhibitors. The findings reveal a noteworthy potential in the development of antiviral agents for CoVs. Moreover, the fungal bioactive compounds that possess immunomodulatory activities reveal themselves to be a potential resource in the treatment of CoVs.

## 3. Potential of Fungal Antiviral Bioactive Compounds as Protease Inhibitors to Treat CoVs

Fungi (including filamentous fungi and mushrooms) represent a rich source of various biologically active compounds that can serve as a major source of new compounds in the development of small-molecule drugs. This development process could involve direct or semi-synthetic methods, while the findings of this research could serve as a source of inspiration in the investigation of chemical scaffolds. Bioactive compounds that were obtained from fungi with potent antiviral activity are presently under investigation, and the number of studies is continually increasing [25,26,27,28,29,30]. Fungal bioactive compounds can be divided into two major groups of molecules; small organic molecules (secondary metabolites) produced by filamentous fungi, especially endophytic fungi, and high molecular weight compounds in the extracts or products that were obtained from the fruiting bodies of edible or medicinal mushrooms [26,31,32,33]. Fungal small organic molecules are low molecular weight compounds that are produced by filamentous fungi. These compounds were synthesized by fungal hyphae and later secreted. They are commonly studied through the cultivation of fungal hyphae in culture media. Small molecular weight compounds with antiviral capabilities have been classified as indole alkaloids, non-ribosomal peptides, polyketides, and hybrids of non-ribosomal peptides and polyketides, and terpenoids [24,25,30,31,33,34,35,36,37,38]. The antiviral activity of high molecular weight compounds that are extracted from fruiting bodies and fungal mycelia have been reported and classified as lignin derivatives, polysaccharides (e.g. chitin, glucan, lentinan, and mannan), proteins, and polysaccharide-protein/amino acid complexes [28,29,30,39,40,41,42]. Drugs or compounds with special effects on viral protease inhibitors, like HIV-1 protease inhibitors and hepatitis C virus (HCV) NS3/4A protease inhibitors, have been considered as potential drugs against CoVs, according to the findings of previous studies. Therefore, fungal compounds that have the potential to be candidates as protease inhibitors have been the focus of numerous present studies.

**Figure 2:**
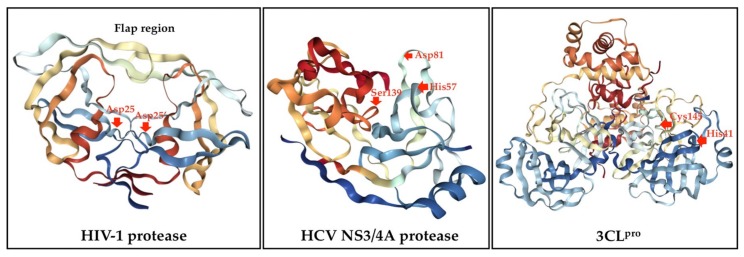
Crystal structures of HIV-1 protease (PDB: 2NMZ), HCV NS3/4A protease (PDB: 1DY8) and 3CL^pro^ (PDB: 2DUC). The catalytic sites are arrowed.

The major viral protease classes have been identified based on the relevant catalytic types including serine, cysteine, aspartic, threonine, glutamic acid, and metalloproteases [43,44]. Most viral proteases can recognize the specific sequences of amino acids in their substrates and cleave the peptide bond via a nucleophilic attack on the side chain of catalytic site [43,44,45]. HIV-1 protease is formed by two identical monomers as shown in Figure 2. Each monomer contributes on catalytic aspartyl residues (Asp25 and Asp25ʹ) in the active site which lie on the bottom of the cavity that plays a crucial role in substrate binding [46]. Additionally, HCV polyprotein is processed proteolytically upon translation by both host cells and viral proteases to at least 10 individual proteins [47,48]. These include four structural proteins and non-structural (NS) proteins. NS3/4A serine protease is further involved in the proteolytic processing of NS proteins and is also considered necessary for the direct-inhibition of HCV. NS3 is comprised of protease and helicase domains and forms a heterodimer with NS4A. Additionally, NS4A binds to the N-terminal region of NS3 and acts as a cofactor of the protease to enhance cleavage (Figure 2). The catalytic triad of the NS3/4A protease is formed by His57, Asp81, and Ser139 [47,48,49]. Currently, the NS3 protein has emerged as an important target for anti-HCV drug discovery and development. Notably, a 3CL^pro^ of CoVs is comprised of three-domain cysteine proteases. Furthermore, domain I and II contain β-barrels of the chymotrypsin structure, but domain III consists mainly of α-helices (Figure 2). Moreover, 3CL^pro^ contains a catalytic dyad defined by His41 and Cys145 [50,51]. This main protease is responsible for maturation of functional proteins and currently represents as a key target for antiviral drugs. Therefore, most of antiviral agents are peptidomimetics and macrocyclic compounds that interact with the active site of a targeted viral protease [52,53]. According to the HIV-1 protease and HCV protease exhibited a similar function as CoVs protease, so protease inhibitors are hypothesized to have the preventive and therapeutic potential against CoVs infection.

### 3.1. HIV-1 Protease Inhibitors Isolated from Fungi

Several HIV-1 protease inhibitor drugs have been made available in the human clinical use of CoVs [4,7,8,16,17,18]. Nelfinavir was found to strongly inhibit the replication of SARS-CoV [54,55]. Antiviral drugs (ribavirin, lopinavir, and ritonavir), steroids, proteins that are known as immunoglobulins, type I interferon, and convalescent plasma have been used in the clinical treatment of SARS and MERS patients [56,57,58,59,60]. A diagnostic test for early SARS and MERS illnesses has not been validated; therefore, treatment could only be initiated once patients have met the criteria of a clinical and epidemiological case definition. Patient characteristics, such as age and the presence of diabetes, have been associated with severe diseases and they can confound treatment effects. Certain potential drugs that have been approved by the FDA and identified as potential inhibitors of 3CL^pro^ of SARS-CoV-2 have been reported by Hosseini and Amanlou [11] while using a virtual screening and the molecular docking procedure. The ten potential drugs include paclitaxel, simeprevir, docetaxel, palbociclib, cabazitaxel, alctinib, imatinib, plerixafor, azelastine, and dasabuvir. Paclitaxel and simeprevir HCV NS3/4A protease inhibitors) revealed a strong degree of interaction with the SARS-CoV-2 protease binding pocket and it has been placed well into the pocket when compared to the antiviral drugs. Interestingly, virtual screening has confirmed that indinavir was selected as the SARS-CoV-2 main protease (PDB code 6LU7).

**Figure 3:**
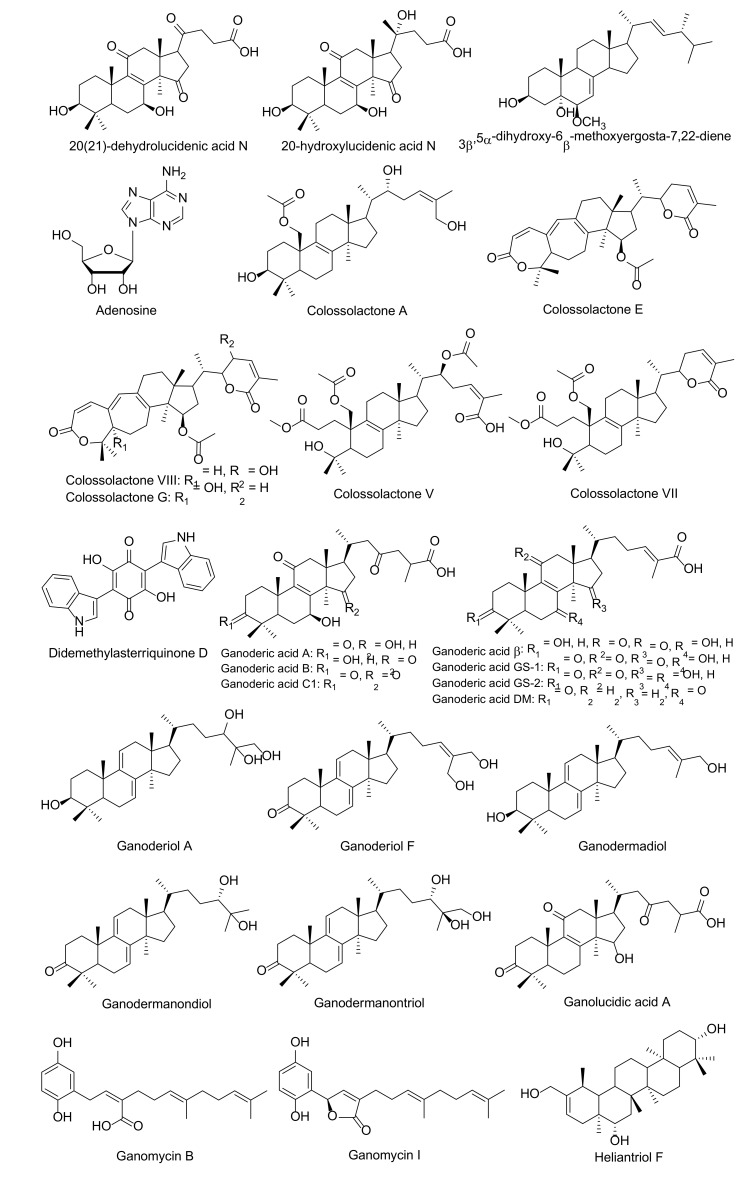

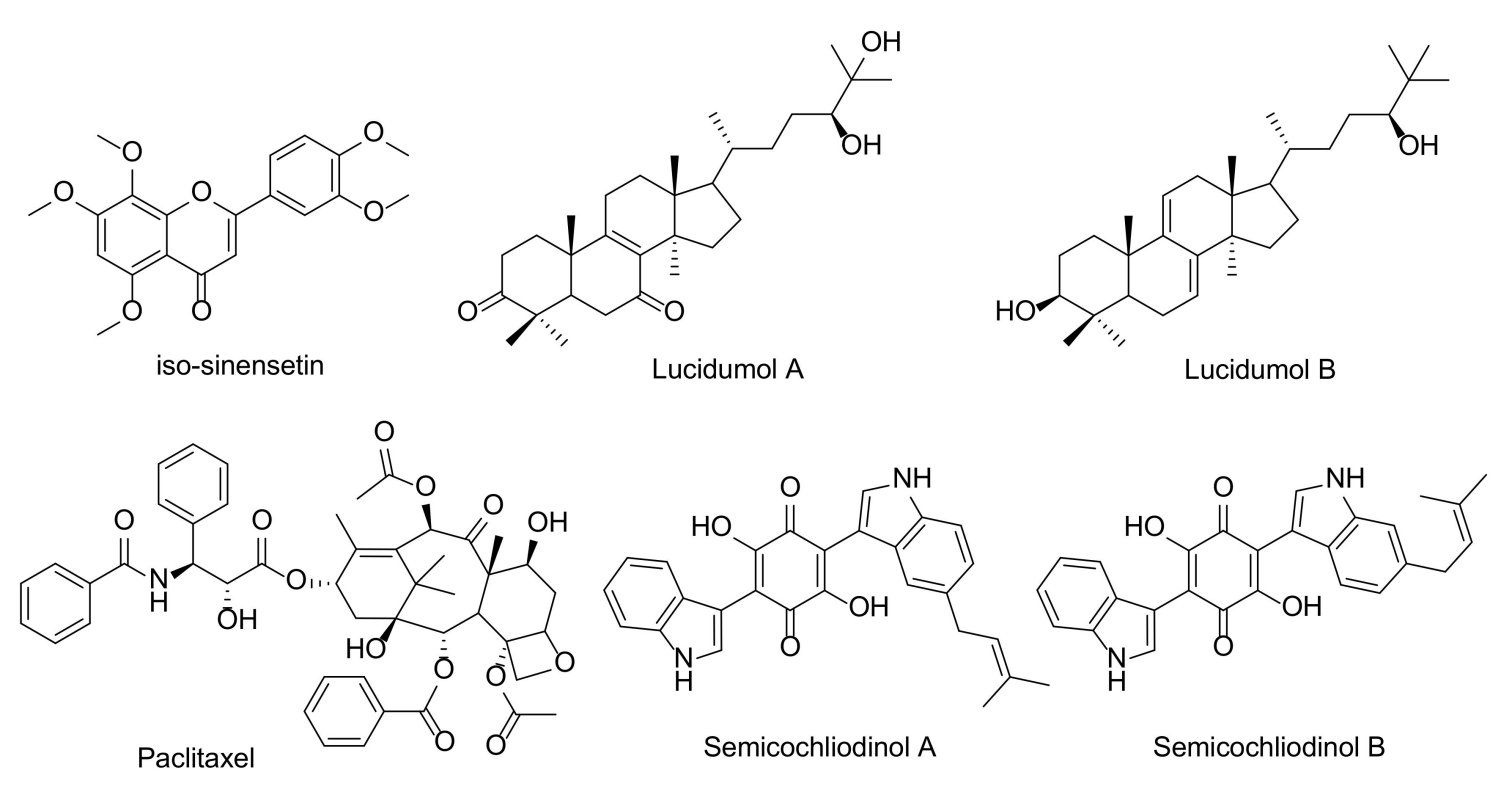
Fungal bioactive compounds for inhibition of HIV-1 protease activity.

HIV-1 protease inhibition has been most thoroughly tested by purified and unpurified fungal metabolites. Table 1 and Figure 2 show fungal bioactive agents for HIV-1 protease inhibitors are shown in Table 1 and Figure 3. Interestingly, paclitaxel or taxol, a chemotherapeutic diterpenoid natural compound, was first extracted from the bark of trees that belong to the genus *Taxus*. This compound was produced by several endophytic fungi in the genera *Alternaria*, *Aspergillus*, *Beauveria*, *Cladosporium*, *Chaetomella*, *Fusarium*, *Guignadia*, *Monochaetia*, *Nodulisporium*, *Pestlotia*, *Pestalotiopsis*, *Pithomyces*, *Penicillium*, *Phomopsis*, *Phyllostica*, *Sporormia*, *Taxomyces*, *Trichoderma*, *Trichothecium*, *Tubercularia*, and *Xylaria* [61,62,63,64,65,66,67,68,69]. More than sixty endophytic fungal strains have been identified as paclitaxel producers [70,71]. Generally, paclitaxel has been used as an anticancer drug against breast cancer, non-small cell lung cancer, ovarian cancer, and prostate cancer [72,73]. However, paclitaxel is now being considered for its inhibitory effect on HIV-1 protease activity.

Ryang et al. [74] reported that 20 μg/mL of paclitaxel could inhibit HIV-1 protease activity in a similar manner to the positive control pepstatin A (80 μg/mL) in the in vitro experiment. A combination of paclitaxel and protease inhibitors (indinavir, nelfinavir, or combinations of these agents) at recommended dosages and schedules was used to treat patients of HIV-associated Kaposi’s Sarcoma without enhancing toxicity [75]. In the virtual screening procedure, paclitaxel has been suggested as a therapeutic agent of SARS-CoV-2 based on its higher binding energy (–11.33 kcal/mol) to the active site of SARS-CoV-2 protease than that of lopinavir (–5.36 kcal/mol) and ritonavir (–5.04 kcal/mol) [21]. However, patient conditions for paclitaxel applications should be considered because of its side effects on bone marrow suppression. In addition, two semicochliodinols (semicochliodinol A and B) and didemethylasterriquinone D that were isolated from a microfungus, *Chrysosporium merdarium*, displayed an inhibitory effect on HIV-1 protease activity [76,77].

Some HIV-1 protease inhibitors have been isolated from certain mushrooms, especially some edible and medicinal mushrooms. Lingzhi mushrooms (*Ganoderma* species) have been generally acknowledged as a nutritional supplement across the world due to their association with long-term safety and the fact that they possess a vast array of medicinal properties. Various compounds that have exhibited inhibitory effects against HIV-1 protease activity have been identified from *Ganoderma lucidum* including ganolucidic acid A, 3β-5α-dihydroxy-6β-methoxyergosta-7,22-diene, ganoderic acid A–C, ganoderic acid β, ganodermanondiol, ganodermanontriol and lucidumol B [78,79,80]. Six colossolactones, ganomycin I, and ganomycin B isolated from *G. colosum* have displayed anti-HIV-1 protease activity [81,82]. Twenty-five metabolites were isolated from the fruiting body of *G. sinnense*, and it was found that ganoderic acid GS-2, 20-hydroxylucidenic acid N, 20(21)-dehydrolucidenic acid N and ganoderiol F exhibited a high potential to inhibit HIV-1 protease activity [83]. Notably, crude extracts of tiger milk mushroom (*Lignosus rhinocerus*) displayed inhibitory activity against HIV-1 protease activity on infected cells, while in silico analysis showed that heliantriol F displayed significant binding energy at -12.57 kcal/mol on the active site of HIV-1 protease [84]. Hexane extract fractions obtained from a jelly fungus (*Auricularia polytricha*) could effectively inhibit HIV-1 protease activity in vitro, while four major compounds, ergosterol, linoleic acid and two triacylglycerols were found to be present [85]. Moreover, adenosine and iso-sinensetin isolated from golden cordycep (*Cordycep militaris*), and 4.5 kDa protein isolated from *Russula paludosa*, have been reported as anti-HIV-1 replications by inhibition of HIV-1 protease activity [86,87].

### 3.2. HCV NS3/4A Protease Inhibitors Isolated from Fungi

**Figure 4:**
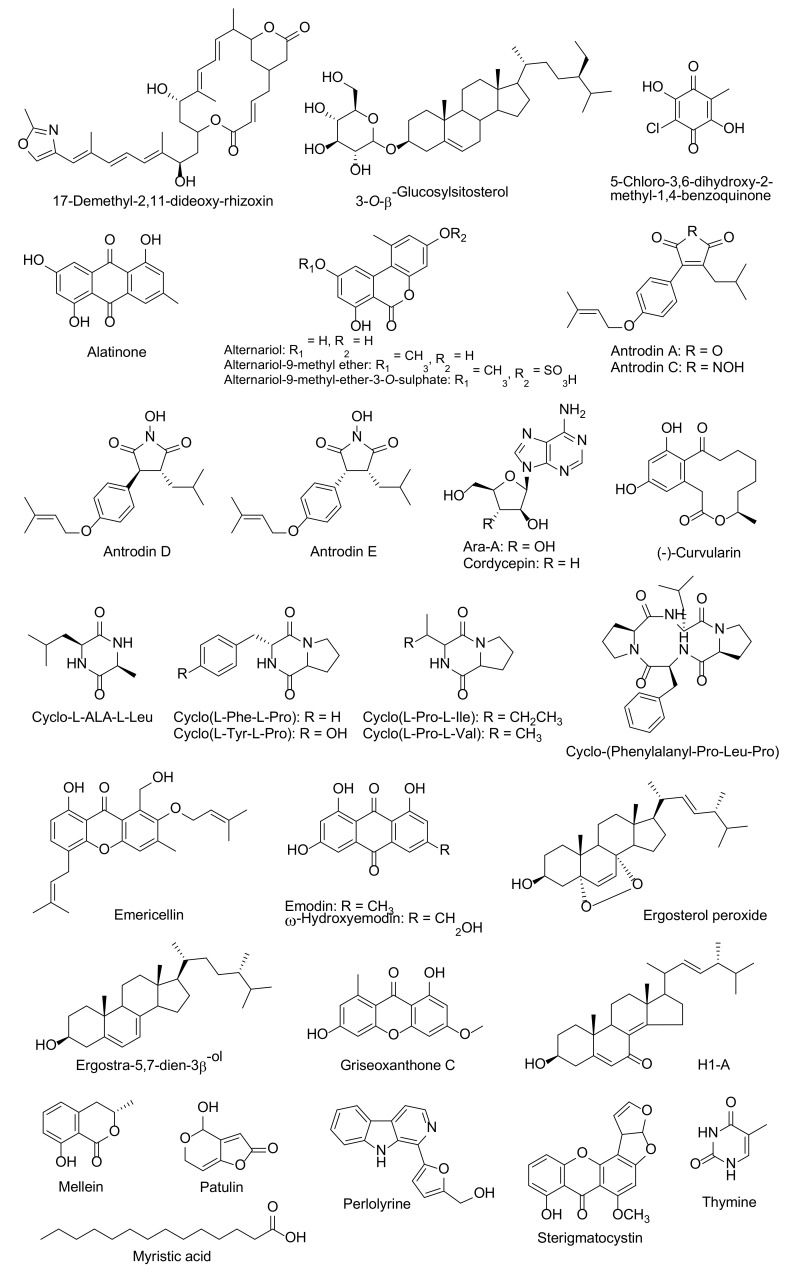
Fungal bioactive compounds for inhibition of HCV NS3/4A protease.

Simeprevir, which is a HCV NS3/4A protease inhibitor, has been acknowledged as a highly effective agent of SARS-CoV-2 that can display a higher energy value (–11.33 kcal/mol) for the binding active site of SARS-CoV-2 protease than lopinavir and ritonavir [20]. However, there has been an absence of clinical test support for this outcome. Additionally, patient conditions for simeprevir applications should be considered, because it can commonly cause a rash, nause, and muscle pain, as well as an allergic reaction [11]. In published literature, there are several bioactive compounds isolated from endophytic fungi and mushrooms that have been identified for the inhibition of HCV NS3/4A protease (Table 2 and Figure 4). An aqueous extract with a low molecular weight (< 3 kDa) fraction of the white button mushroom (*Agaricus bisporus*) has displayed a responsible activity to the indictors of HCV replication [88]. Alternaroil and alternariol derivatives (alternariol-9-methyl ether-3-*O*-sulphate and alternariol-9-methyl ether) of an endophytic fungus, *Alternaria alternate*, and their metabolites were explored for the inhibition of HCV NS3-NS4A protease [89,90,91,92]. Hawas et al. [93] found that the most potent HCV NS3/4A protease isolated compound that was obtained from *Fusarium equiseti* were ω-hydroxyemodin and Griseoxanthone C. Furthermore, mellein, patulin, and H1-A were isolated from *Aspergillus ochraceus*, *Penicillium griseofulvum*, and *Fusarium oxysporum*, respectively. These compounds displayed activity against HCV NS3/4A protease [94,95,96]. *Antrodia cinnamomea*, a medicinal mushroom, produced antrodins A–E. Antrodins A showed potent inhibitory capabilities of HCV protease activity [97]. Five products that were obtained from the endophytic fungus *Emericella nidulans*, namely cordycepin, emericellin, ergosterol peroxide, myristic acid, and sterigmatocystin, reported having HCV NS3/4A protease inhibitory properties [98,99,100,101,102]. Moreover, Ahmed et al. [103] isolated the metabolite compounds for HCV NS3/4A protease inhibitors that were obtained from *Aspergillus versicolor* that possess constituents of (−)-curvularin, cyclo(L-Pro-L-Ile), cyclo(L-Tyr-LPro), cyclo(L-Phe-L-Pro), cyclic tetrapeptide, and cyclo-(Phenylalanyl-pro-Leu-pro). Three metabolites were isolated from an endophytic fungus, *P. chrysogenum* [104]. These compounds were identified as alatinone, emodin, and ω-hydroxyemodin, and they displayed activities against HCV NS3/4A protease.

## 4. Potential of Fungal Bioactive Compounds for Immunomodulators

Inflammasome is a cytosolic multiprotein oligomer of the innate immune system that is responsible for the activation of inflammatory responses. Inflammasome induction by coronavirus was first reported in porcine reproductive and respiratory syndrome virus [105]. Currently, the transport of Ca^2+^ by SARS-CoV has been reported to trigger inflammasome activation. It has been suggested that the cytokine storm is associated with cases of pneumonia that were infected by SARS-CoV-2 [106]. Cytokines and chemokines have been recognized for playing an important role in immunity and immunopathology in the body during virus infection. They are an important part of the first barrier of innate immunity that serves as a defense against the viruses. The massive infiltrated inflammatory cells and the elevated proinflammatory cytokines/chemokines can lead to fatal acute lung injury (ALI) and acute respiratory distress syndrome (ARDS) [107,108]. A clinical study of 41 patients infected with SARS-CoV-2 in Wuhan, China showed that 63% of the patients had lymphopenia, 12% had ARDS, all patients had pneumonia, and the intensive care patients reported higher plasma levels of IL-2, IL-7, IL-10, GSCF, IP10, MCP1, MIP1A, and TNF-α than the non-intensive care patients [108]. Researchers also noted that patients with high concentrations of IL-1β, IFN-γ, IP10, and MCP1 were likely associated with activated T-helper-1 (Th1) cell responses.

Immunomodulators are the bioactive substances that can play a role or affect the regulating of the immune system, which is the first barrier against infectious diseases [109]. Clinically, immunomodulators are usually classified into three categories based on their relevant activities including: (1) reducing the stimulation of the immune system or reducing the effectiveness of the immune system (immunosuppressants), (2) promoting the response of the innate immune system (immunostimulants), and (3) enhancing the efficacy of vaccines to stimulate immunity (immunoadjuvants) [109,110]. Many drugs are known to be immunomodulatory substances because they have significant clinical efficacy for altering host responses in the therapy of viral and bacterial infections [111,112,113,114]. Various edible mushrooms have been studied for many years in terms of the effects of their metabolites in boosting immune responses and treating infectious [115,116,117,118]. The principal immunomodulatory effect of active substances derived from mushrooms is to stimulate immune effector cells such as T cells, cytotoxic T lymphocytes (CTL), dendritic cells (DCs), lymphocytes, macrophages and natural killer (NK) cells, resulting in cytokine expression and secretion including interleukins (ILs), tumor necrosis factor-alpha (TNF)-α, and interferon-gamma (INF)-γ [119,120].

Immunomodulators derived from mushrooms are classified into four groups, including lectins, proteins, polysaccharides, and terpenoids [109]. Lectins are carbohydrate-binding proteins that can be found in many organisms and are extracted from mushrooms. They have specific immune cell functions such as antiproliferative, and antitumor activities [108].

**Figure 5:**
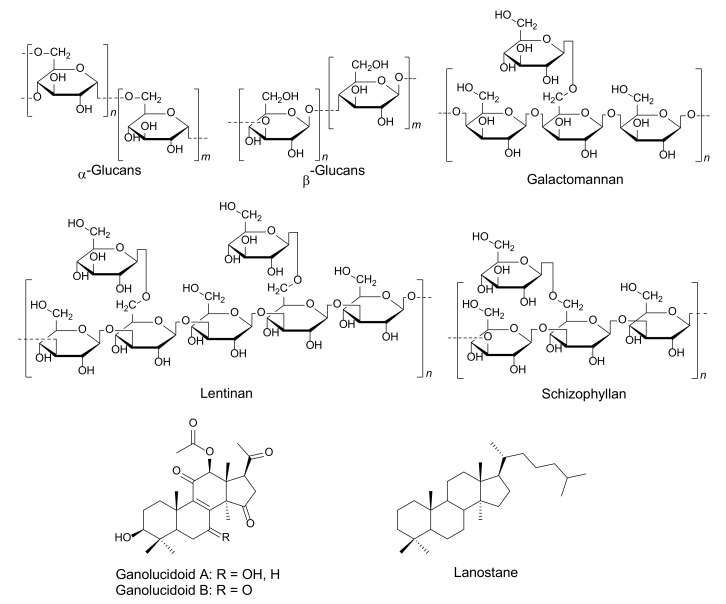
Fungal bioactive compounds for immunomodulators.

Fungal immunomodulatory proteins (FIPs) are small molecular weight proteins, ∼13 kDa and 110–114 amino acids, displaying immunomodulatory activity. They are a type of bioactive substance that can be derived from some edible mushrooms. Meanwhile, mushrooms are an essential source of immunomodulatory polysaccharides, which are long chains of carbohydrate molecules, particularly polymeric carbohydrates, that are composed of monosaccharides linked together by glycosidic bonds [108]. Polysaccharides are responsible for immuno-modulating activities that include stimulating phagocytic activity, acting as inflammatory mediators and in cytokine production [121,122,123]. Terpenes and terpenoids are a large and diverse class of hydrocarbon compounds and typically consist of five-carbon isoprene units [109,124]. Many terpenoids are biologically active and have been widely used for the treatment of many diseases. Simultaneously, they play a diverse role in the fields of cosmetic and food production and have been associated with hormones, medicines, vitamins, etc. [124]. Triterpenoids such as lanostane are the highly oxidized substances that can be isolated from wood-decaying mushrooms, *Ganoderma* sp. These compounds display immunomodulating and anti-infective effects [125,126,127]. Many species of mushrooms have been found to produce immunomodulators, such as *Agaricus bisporus*, *Agaricus blazei*, *Amanita pantherina*, *Boletus satanas*, *Coprinus cinereus*, *Cordyceps sinensis*, *Ga. lucidum*, *Grifola frondosa*, *Flammulina velutipes*, *Ischnoderma resinosum*, *Lactarius deterrimus*, *Laetiporus sulphureus*, *Lentinus tigrinus*, *Trametes versicolor*, and *Volvariella volvacea* [115,128,129,130], as is detailed in Figure 5 and Table 3.

The fungal immunomodulatory protein FIP-fve that was obtained from *Flammulina velutipes* has been employed to suppress the respiratory syncytial virus (RSV), which is known to cause bronchiolitis. FIP-fve effectively decreased RSV replication, IL-6 expression, and inflammation via inhibition of NF-κB translocation and respiratory pathogenesis in RSV-challenged mice. Interestingly, FIP-fve maymight be seen as a safe substance for viral prevention and disease therapy [133]. Immunomodulators have become useful agents in relieving the pathology that is associated with viral infections going forward [152]. The immunomodulatory mechanisms of mushroom products involve stimulating innate and adaptive immune responses through the activation of macrophages, T lymphocytes, DCs, NK cells, and cytokines. A study of the relationship between the structure and activity of immunomodulators will encourage the development of new therapeutic agents for the treatment of viral infection diseases.

## 5. Conclusions

The discovery and production of antiviral metabolites from fungi have emerged as part of an exciting field in viral therapeutic and antiviral drug development. Although, CoVs vaccines have been continually developed to alter the occurrence of virally associated diseases, viral protease inhibitors and immunomodulators have become extremely useful agents in this process. The results of the current studies indicate that fungi are an important source of the natural bioactive compounds that have potential as protease inhibitors and immunomodulations. Fungal protease inhibitors reveal strong potential as future candidates in the development of antiviral drugs or alternative and complementary medicals prevention and treatment of CoVs. However, it is of particular interest and concern that fungal protease inhibitors and fungal extracts could have both poisonous and curative effects against CoVs. Presently, there has been a lack of clinical tests that can validate these determinations. Consequently, these circumstances may result in consumers delaying or stopping their pursuit of appropriate medical treatment, which may lead to serious and life-threatening harm to those individuals. Therefore, laboratory assays and clinical tests are needed to fully understand the level of toxicity and pharmacokinetic profile of these viral protease inhibitors and immunomodulators. The important research must be done before the application of these fungal compounds can be used for the prevention and treatment of CoVs in the future, particularly with regard to SARS-CoV-2.

## Figures and Tables

**Table 1:** Fungal bioactive compounds for HIV-1 protease inhibitors that potential candidate to treat CoVs.

Source	Bioactive Agent	Efficacy*	Reference
Endophytic fungi in genera *Alternaria*, *Aspergillus*, *Beauveria*, *Cladosporium*, *Chaetomella*, *Fusarium*, *Guignadia*, *Monochaetia*, *Nodulisporium*, *Pestlotia*, *Pestalotiopsis*, *Pithomyces*, *Penicillium*, *Phomopsis*, *Phyllostica, Sporormia, Taxomyces*, *Trichoderma*, *Trichothecium*, *Tubercularia* and *Xylaria*	Paclitaxel	20 μg/mL, viral inhibition was similar to positive control pepstatin A (80 μg/mL). CC_50_ > 50 μg/mL in human embryonic kidney 293 (HEK-293) cells	[61,62,63,64,65,66,67,68,69,70,71,74]
*Chrysosporium merdarium*	Semicochliodinol A	IC_50_ = 0.37 μM CC_50_ = 0.84 μM in human lung fibroblast cells	[75,76]
	Semicochliodinol B	IC_50_ > 0.5 μM	[77]
	Didemethylasterriquinone D	IC_50_ = 0.24 μM	[77]
*Ganoderma lucidum*	Ganolucidic acid A	IC_50_ = 70 μM	[78]
	Ganoderic acid A	IC_50_ = 430 μM CC_50_ > 62.5 μM on normal human fibroblast BJ cells	[78,79]
	Ganoderic acid B	IC_50_ = 140 μM	[80]
	Ganoderic acid C1	IC_50_ = 240 μM	[80]
	Ganoderic acid β	IC_50_ = 20 μM	[80]
	Ganodermanondiol	IC_50_ = 90 μM	[80]
	Ganodermanontriol	IC_50_ = 70 μM	[80]
	Lucidumol B	IC_50_ = 50 μM	[80]
	3β-5α-dihydroxy-6β-methoxyergosta-7,22-diene	IC_50_ = 7.8 µg/mL	[80]
	Ganomycin B	IC_50_ = 7.5 µg/mL	[81,82]
*Ganoderma colosum*	Ganomycin I	IC_50_ = 1 µg/mL	[81,82]
	Colossolactone A	IC_50_ = 39 µg/mL	[81]
	Colossolactone E	IC_50_ = 8 µg/mL	[81]
	Colossolactone G	IC_50_ = 5 µg/mL	[81]
	Colossolactone V	IC_50_ = 9 µg/mL	[81]
	Colossolactone VII	IC_50_ = 13.8 µg/mL	[81]
	Colossolactone VIII	IC_50_ = 31.4 µg/mL	[81]
*Ganoderma sinnense*	Ganoderic acid GS-1	IC_50_ = 58 μM	[83]
	Ganoderic acid GS-2	IC_50_ = 30 μM	[83]
	Ganoderic acid DM	IC_50_ = 38 μM	[83]
	Ganoderic acid β	IC_50_ = 116 μM	[83]
	Ganoderiol A	IC_50_ = 80 μM	[83]
	Ganoderiol F	IC_50_ = 22 μM	[83]
	Ganodermadiol	IC_50_ = 29 μM	[83]
	Ganodermanontriol	IC_50_ = 65 μM	[83]
	Lucidumol A	IC_50_ = 99 μM	[83]
	20-hydroxylucidenic acid N	IC_50_ = 25 μM	[83]
	20(21)-dehydrolucidenic acid N	IC_50_ = 48 μM	[83]
*Lignosus rhinocerus*	Heliantriol F	Binding energy −12.57 kcal/mol	[84]
*Auricularia polytricha*	Hexane extract fraction	0.80 ± 0.08 mg/ml	[85]
*Russula paludosa*	4.5 kDa protein	IC_50_ = 0.25 mg/mL	[86]
*Cordycep militaris*	Adenosine	No quantifiable results	[87]
	iso-sinensetin	No quantifiable results	[87]

*IC_50_ = the half maximal inhibitory concentration and CC_50_ = the half maximal cytotoxic concentration.

**Table 2:** Fungal bioactive compounds for HCV NS3/4A protease inhibitor as potential candidates for the treatment of CoVs, particularly SARS-CoV-2.

Source	Bioactive Agent	Efficacy*	Reference
*Agaricus bisporus*	Aqueous extract with low molecular weight (< 3 kDa) faction	20.5 µg/mL, viral inhibition = 67.2–87.7%	[88]
*Alternaria alternata*	Alternariol	IC_50_ = 52.0 ± 4.4 µg/mL IC_50_ = 52.0 ± 4.4 µg/mL CC_50_ > 10 µg/mL on human bronchial epithelial BEAS-2B cells	[89,90,91]
	Alternariol-9-methyl- ether-3-*O*-sulphate	IC_50_ = 32.3 ± 2.6 µg/mL	[89]
	Alternariol-9-methyl ether	IC_50_ = 12.0 ± 3.8 µg/mL CC_50_ > 7.7 µg/mL on human bone osteosarcoma epithelial U-2 OS cells	[89,92]
*Antrodia cinnamomea*	Antrodin A	IC_50_ = 0.9 µg/mL	[97]
	Antrodin C	IC_50_ = 2.9 µg/mL	[97]
	Antrodin D	IC_50_ = 20.0 µg/mL	[97]
	Antrodin E	IC_50_ = 20.1 µg/mL	[97]
*Aspergillus ochraceus*	Mellein	IC_50_ = 35 μM	[96]
*Aspergillus versicolor*	(−)-Curvularin	IC_50_ = 37.5 ± 3.6 µg/mL	[103]
	Cyclo(L-Pro-L-Ile)	IC_50_ = 13.7 ± 3.3 µg/mL	[103]
	Cyclo(L-Tyr-L-Pro)	IC_50_ = 8.2 ± 1.7 µg/mL	[103]
	Cyclo(L-Phe-L-Pro)	IC_50_ = 88.8 ± 4.5 µg/mL	[103]
	Cyclo- (Phenylalanyl-Pro-Leu-Pro)	IC_50_ = 95.3 ± 2.7 µg/mL	[103]
*Emericella nidulans*	Cordycepin	IC_50_ = 24.5 ± 2.3 µg/mL CC_50_ > 3.2 µg/mL on human umbilical vein endothelial cells and > 100 µg/mL on HEK 293 cells	[98,99,100]
	Emericellin	IC_50_ = 50.0 ± 3.8 µg/mL	[98]
	Ergosterol peroxide	IC_50_ = 47.0 ± 3.4 µg/mL CC_50_ 95 µg/mL on normal lung BEAS-2B cells and > 26.7 µg/mL normal human fibroblast BJ cells	[98,101]
	Myristic acid	IC_50_ = 51.0 ± 2.6 µg/mL CC_50_ > 50 µg/mL on human dermal fibroblast cells	[98,102]
	Sterigmatocystin	IC_50_ = 48.5 ± 4.2 µg/mL	[98]
*Fusarium equiseti*	Griseoxanthone C	IC_50_ = 19.88 ± 1.45 μM	[93]
	ω-Hydroxyemodin	IC_50_ = 10.7 μM	[93]
	Cyclo-L-ALA-L-Leu	IC_50_ = 58.33 ± 3.51 μM	[93]
	Cyclo(L-Pro-L-Val)	IC_50_ = 23.29 ± 1.23 μM	[93]
	Thymine	IC_50_ = 51.82 ± 2.49 μM	[93]
	Cyclo-(Phenylalanyl-Pro-Leu-Pro)	IC_50_ = 29.45 ± 1.98 μM	[93]
	17-Demethyl-2,11-dideoxy-rhizoxin	IC_50_ = 34.42 ± 1.44 μM	[93]
	Ergostra-5,7-dien-3β-ol	IC_50_ = 77.14 ± 4.55 μM	[93]
	3-*O*-β-Glucosylsitosterol	IC_50_ = 76.56 ± 3.78 μM	[93]
	5-Chloro-3,6-dihydroxy-2-methyl-1,4-benzoquinone	IC_50_ = 35.15 ± 3.92 μM	[93]
	Cyclo(L-Tyr-L-Pro)	IC_50_= 18.20 ± 1.7 μM	[93]
	Perlolyrine	IC_50_ = 37.89 ± 2.11 μM	[93]
	Cordycepin	IC_50_ = 22.35 ± 3.12 μM CC_50_ > 3.2 µg/mL on human umbilical vein endothelial cells and > 100 µg/mL on HEK 293 cells	[93]
	Ara-A	IC_50_ = 24.53 ± 2.3 μM	[93]
*Fusarium oxysporum*	H1-A	VX950 inhibitory constant value was 3.5 μmol/L	[94]
*Penicillium chrysogenum*	Alatinone	IC_50_ = 370 μM	[104]
	Emodin	IC_50_ = 80 μM	[104]
	ω-Hydroxyemodin	IC_50_ = 30 μM	[104]
*Penicillium griseofulvum*	Patulin	IC_50_ = 24.7 µM	[95]

*IC_50_ = the half maximal inhibitory concentration and CC_50_ = the half maximal cytotoxic concentration.

**Table 3:** Immunomodulatory activities of mushrooms.

Category	Bioactive Agent	Source	Immune Effects	Reference
Lectins	Concanavalin A	*Volvariella volvacea*	Activating T lymphocytes	[130]
	Ricin-B-like lectin (CNL)	*Clitocybe nebularis*	Stimulating dendritic cells (DCs) and cytokines	[131]
	TML-1, TML-2	*Tricholoma mongolicum*	Macrophages activator (TNF-α, Nitrite ions)	[132]
Fungal immunomodulatory proteins (FIPs)	FIP-fve	*Flammulina velutipes*	Stimulating lymphocyte mitogenesis, enhancing transcription of IL-2, IFN- γ, and TNF-α	[133,134]
	Fip-gat	*Ganoderma atrum*	Inducing apoptosis via autophagy	[135]
	Fip-gts	*Ganoderma tsugae*	Inducing apoptosis via autophagy	[136]
	FIP-gsi	*Ganoderma sinensis*	Cytokines regulation (IL-2, IL-3, IL-4, IFN- γ, TNF-α)	[137]
	Fip-lti1, Fip-lti2	*Lentinus tigrinus*	Cytokines regulation (TNF-α, IL-1β, and IL-6)	[138]
	FIP-ppl	*Postia placenta*	Enhancing interleukin-2 (IL-2)	[139]
	FIP-SJ75	*Ganoderma lucidum*, *Flammulina velutipes*, *Volvariella volvacea*	Activating macrophage M1 polarization and initiating pro-inflammatory response	[121]
	Fip-vvo	*Volvariella volvacea*	Lymphocytes activator, cytokine regulation	[140]
	GMI	*Ganoderma microsporum*	Inducing apoptosis via autophagy	[141]
	Ling Zhi-8 (Lz-8)	*Ganoderma lucidum*	T cell and macrophages activator, cytokine regulation	[142,143]
Polysaccharides	α- and β-glucans	*Agaricus bisporus*, *Agaricus brasiliensis*, *Ganoderma lucidum*	Inducing synthesis of IFN-γ	[144]
	β-glucan	*Grifola frondosa*	Activating macrophages, NK cells, lymphokines and cytokines	[145,146]
Polysaccharides	Galactomannan	*Morchella esculenta*, *Morchella conica*	Activating macrophages and cytokines	[147,148]
	Grifolan	*Grifola frondosa*	Activating macrophages and lymphokines	[149]
	Lentinan	*Lentinus edodes*	T-cell-oriented adjuvant	[149]
	PS-G	*Ganoderma lucidum*	Activating macrophages and T lymphocytes	[135,136]
	Schizophyllan	*Schizophyllum commune*	Activating T cell, increasing interleukin and TNF-α production	[150]
Terpenoids	Exobiopolymers	*Ganoderma applanatum*	Activating NK cell	[128]
	Ganolucidoid A and B	*Ganoderma lucidum*	NO production, anti-inflammatory activities	[130]
	Lanostane	*Hypholoma fasciculare*	NO production, anti-inflammatory activities	[151]
